# Whole-process 3D ECM-encapsulated organoid-based automated high-throughput screening platform accelerates drug discovery for rare diseases

**DOI:** 10.1093/lifemedi/lnaf021

**Published:** 2025-06-14

**Authors:** Zhaoting Xu, Hui Yang, Yuru Zhou, Emmanuel Enoch Dzakah, Bing Zhao

**Affiliations:** State Key Laboratory of Genetic Engineering, School of Life Sciences, Fudan University, Shanghai 200438, China; Institute of Organoid Technology, Kunming Medical University, Kunming 650500, China; School of Basic Medical Sciences, Institute of Biomedical Innovation, The First Affiliated Hospital, Jiangxi Medical College, Nanchang University, Nanchang 330031, China; Z Lab, bioGenous BIOTECH, Shanghai 200438, China; Institute of Organoid Technology, Kunming Medical University, Kunming 650500, China; School of Basic Medical Sciences, Institute of Biomedical Innovation, The First Affiliated Hospital, Jiangxi Medical College, Nanchang University, Nanchang 330031, China; Z Lab, bioGenous BIOTECH, Shanghai 200438, China

**Keywords:** high-throughput screening, organoids, extracellular matrix, neuroendocrine cervical cancer, Quisinostat 2HCl

## Abstract

Organoid-based high-throughput screening (HTS) is revolutionizing pharmaceutical development. However, the complexity of handling extracellular matrix (ECM) components with traditional HTS devices leads to the use of suspension cultures for organoids during HTS, which alters their transcriptomic landscape and drug responses. Although automated generation techniques for 3D ECM-encapsulated organoids have been established, limitations in operational simplicity and time efficiency remain barriers to achieving high throughput. Here, we develop a whole-process 3D ECM-encapsulated organoid-based automated HTS (wp3D-OAHTS) platform, which achieves superior throughput compared to existing reported systems for 3D organoid drug screening. Utilizing this automated platform, we generated more than 10,000 homogeneous 3D organoid domes of neuroendocrine cervical cancer (NECC) and evaluated their drug responses to 2802 compounds in 13 days. This highly efficient and reproducible approach finally enabled the identification of 5 top hits that significantly inhibited NECC organoids *in vitro* with half-maximal inhibitory concentration (IC_50_) of lower than 10 nM. The representative candidate, Quisinostat 2HCl, demonstrated significantly stronger anti-tumor efficacy than clinically used agents *in vivo*. This platform significantly improves the rapidity and efficiency of 3D ECM-encapsulated organoid drug screening and facilitates new drug discovery for rare diseases.

## Introduction

The application of organoids for *ex vivo* high-throughput screening (HTS), particularly patient-derived organoids (PDOs), has gained widespread acceptance in drug development and precision medicine [1–5]. HTS allows for the rapid assessment of the sensitivity and resistance of thousands of drugs based on their cellular responses, independent of a full understanding of the molecular vulnerabilities underlying specific diseases. This process is advantageous in enabling the quick discovery of treatments for rare diseases lacking genomic mutation information [6]. Compared to conventional two-dimensional (2D) cell lines, organoids with a three-dimensional (3D) structure are more complex, closer to the architecture and behavior of native tissues, and more accurate in predicting drug responses [7]. Compared to patient-derived xenograft (PDX) models, PDOs eliminate the confounding variables associated with interspecies differences and are more cost-effective, time-saving, and resource-efficient.

Organoids are typically developed from stem cells or progenitor cells encapsulated in extracellular matrix (ECM), which is crucial for the establishment of tissue-like architectures, cell–cell communications, and cell–ECM interactions [8–11]. However, previous attempts to employ organoids for HTS have frequently utilized matrix-free or matrix-low suspension culture conditions [1, 3, 12], due to the challenges encountered by traditional automated HTS platforms when handling 3D ECM-encapsulated organoids. On one hand, the viscosity of most ECM components is temperature-dependent, meaning that cell–matrix mixtures will rapidly solidify within the pipette tips when exposed to room temperature during handling by traditional liquid dispensers, thus hindering the dispensing process [13, 14]. On the other hand, the requirement for cell-matrix mixtures to be centrally located in wells poses a significant obstacle when using high-density microplates, such as 96-well or 384-well plates, where well dimensions are exceedingly small. Therefore, organoid cultures were twisted to adapt organoid screenings to the conventional liquid dispenser-based HTS platforms, where the cells are suspended in matrix-low conditions rather than encapsulated in 3D ECM (Fig. S1A). However, even short-term suspension of organoids can lead to changes in their transcriptomic profiles (Fig. S1B and S1C), including the expression of tumor markers (Fig. S1D) and key signaling pathways (Fig. S1E), which may affect drug sensitivity among tumor cells.

While several methods have been developed for the automated generation and drug screening of 3D ECM-encapsulated organoids [15–24], shortcomings in the operational simplicity and time efficiency of these methods hindered their use for large-scale applications. To address the limitations in suspended organoid-based HTS and current 3D organoid-based HTS, we developed a whole-process 3D ECM-encapsulated organoid-based automated HTS (wp3D-OAHTS) platform. This platform can generate large-scale, uniform 3D organoids and perform automated organoid culturing, monitoring, and screening with significantly shorter processing time. Our platform offers superior stability, homogeneity, and reproducibility in 3D ECM-encapsulated organoid cultures. Employing this platform, we conducted a 3D organoid-based HTS of 2802 small molecules against neuroendocrine cervical cancer (NECC) and identified 7 compounds with half-maximal inhibitory concentration (IC_50_) in the low nanomolar range. The representative candidate, Quisinostat 2HCI, was found to have a more effective suppression of NECC xenograft growth compared to the currently used clinical drugs. Moreover, we found that drug screening based on suspended organoids is less accurate in reflecting organoid drug responses compared to using 3D organoids. This proof-of-concept screening demonstrated the potential of this platform for novel drug discovery, especially for rare diseases.

## Results

### An automated HTS platform for 3D ECM-encapsulated organoid generation and drug screening

**Figure 1.**
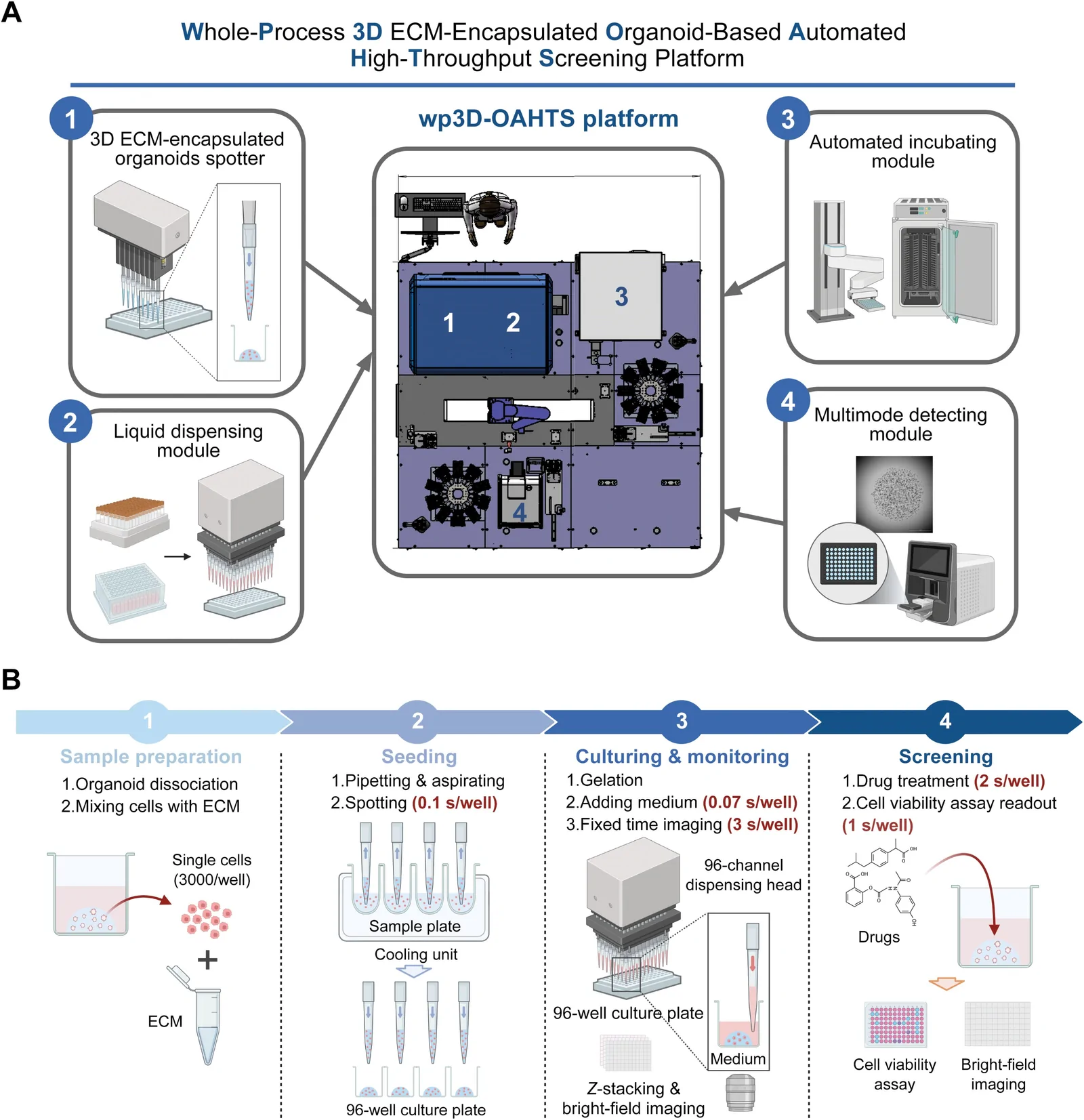
Whole-process 3D ECM-encapsulated organoid-based automated high-throughput screening (wp3D-OAHTS) platform. (A) The top view design and four key modules of the wp3D-OAHTS platform. (B) Workflow of the 3D organoid-based drug screening using wp3D-OAHTS platform.

The standard protocol for organoid culture involves encapsulating dissociated organoids into ECM droplets. However, this culture format is rarely adapted for automated HTS as traditional automated systems are unable to achieve rapid ECM aspiration, precise dispensing, and accurate spatial positioning. Even when implemented in drug screening, this method suffers from limited throughput and high variability in organoid growth. To overcome these restrictions, we developed the wp3D-OAHTS platform, a versatile system compatible with diverse organoid types. This platform integrates a 3D ECM-encapsulated organoids spotter, a liquid dispensing module, an automated incubating module, and a multimode detecting module (Fig. 1A). This system encapsulates and maintains organoids in 80% (*v*/*v*) ECM and enables large-scale and high-speed drug screening entirely based on 3D ECM-encapsulated organoids.

3D ECM-encapsulated organoid spotter is the key to handling ECM components. After sample preparation, the cell–matrix mixtures are added into the sample plate, which is cooled by the cooling unit to prevent the mixtures from solidifying. Before being aspirated, the mixtures in the sample plate are mixed automatically and thoroughly. Pressure sources are used to control the flow rate of cell–matrix mixtures, and the spotting volume is proportional to the plateau time. The homogenized cell–matrix mixtures are then aspirated by an 8-channel ECM spotting head with disposable tips and directly spotted into the wells (3 μL per well) in flying mode, with each well requiring only 0.1 s (Fig. 1B).

Subsequently, the spotted plates undergo gelation on a heating unit until the ECM solidifies completely. A 96-channel liquid dispensing head is then used to add medium into all wells of the plate at once, taking just 0.07 s per well. Next, the plates are transferred by a robotic arm to incubators for culture.

On a daily standardized schedule, the robotic arm transfers the plates from the incubators to the detector for data collection (e.g. imaging), a process that is accomplished in 3 seconds per well, enabling automated continuous monitoring of organoid growth.

On the day of drug administration, the robotic arm retrieves the plates from the incubator and transports them to the liquid dispensing module for medium replacement and drug administration, averaging 2 seconds per well. After 5–7 days of drug treatment, plates are transferred to the liquid dispensing module for medium removal and cell viability assay reagents addition, and then transferred to the incubators for incubation, requiring a scant 2 seconds per well. Finally, the plates are transferred to the detector for cell viability testing, with each well analyzed in only 1 s.

It is estimated that, when operating at full capacity, the wp3D-OAHTS platform can screen 8694 different drugs within a 10-day screening cycle, including 5 days of culturing post-seeding and 5 days of drug treatment. The efficiency and throughput of the platform mark a significant advancement in 3D ECM-encapsulated organoid-based HTS capabilities, demonstrating its potential for expediting drug discovery and personalized medicine.

### Optimized homogeneity and reproducibility of 3D ECM-encapsulated organoid cultures by the wp3D-OAHTS platform

**Figure 2.**
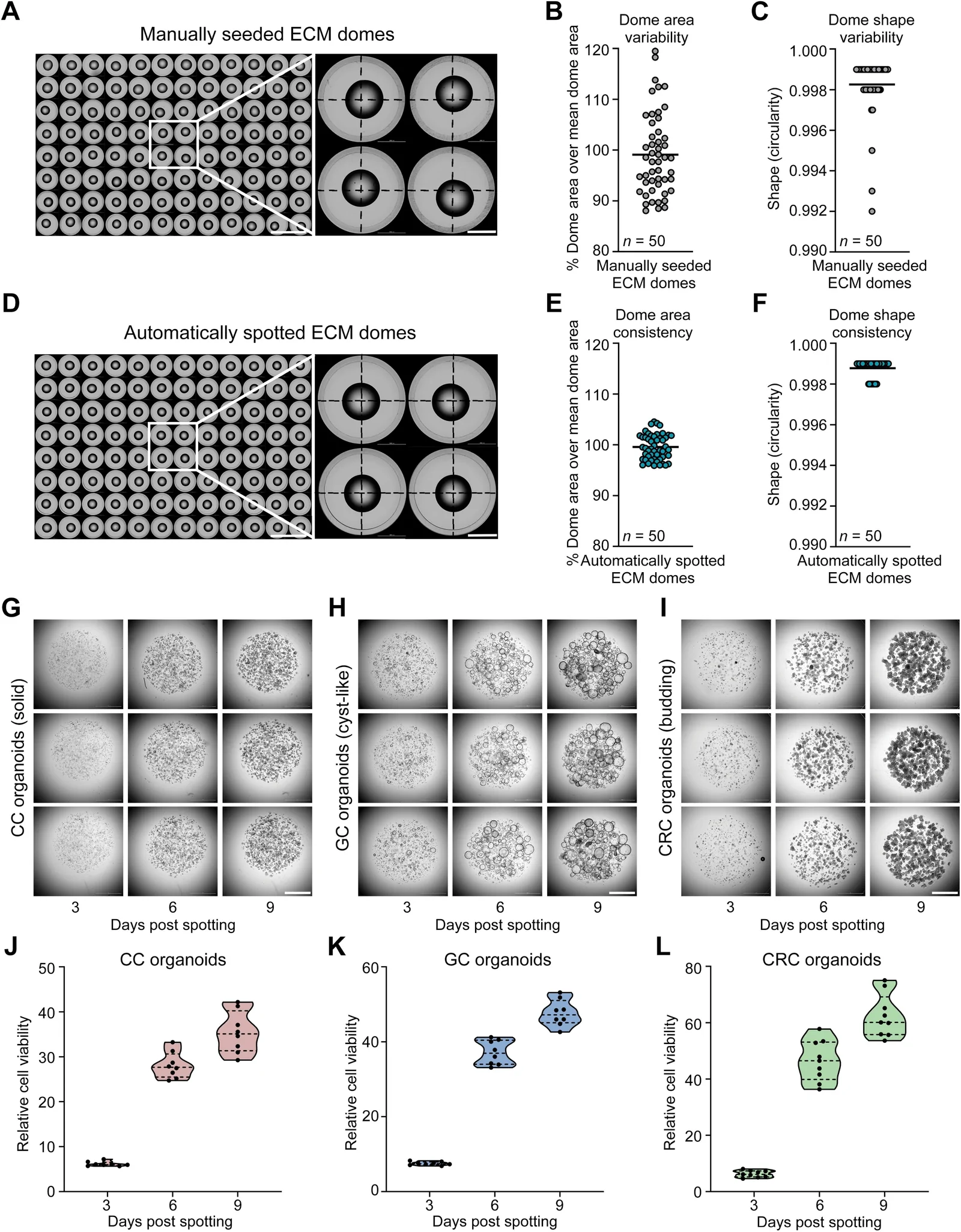
Optimized homogeneity and reproducibility of 3D ECM-encapsulated organoid cultures by the wp3D-OAHTS platform. (A) Overview of an entire 96-well plate filled with manually seeded ECM domes (left) and enlarged view (right). Scale bars, 10 mm (left) and 2 mm (right). (B) Quantification of the dome-to-dome area variability of manually seeded ECM domes (dots). (C) Quantification of the shape (circularity) of manually seeded ECM domes (dots). (D) Overview of an entire 96-well plate filled with automatically spotted ECM domes (left) and enlarged view (right). Scale bars, 10 mm (left) and 2 mm (right). (E) Quantification of the dome-to-dome area variability of automatically spotted ECM domes (dots). (F) Quantification of the shape (circularity) of automatically spotted ECM domes (dots). (G–I) Representative bright-field images showing CC (G), GC (H), and CRC (I) organoids growth after being automatically spotted and cultured by wp3D-OAHTS platform. Scale bar, 1 mm. (J–L) Violin plots of cell viability change ratio of cervical cancer (J), gastric cancer (K), and colorectal cancer (L) organoids after being automatically spotted and cultured by wp3D-OAHTS platform.

A major challenge in establishing 3D ECM-encapsulated organoid-based HTS is that the introduction of ECM significantly increases operational complexity and compositional variability, leading to elevated well-to-well heterogeneity and reduced screening accuracy. Manual seeding further exacerbates operator-to-operator variability and batch-to-batch inconsistencies, while current automated systems remain insufficient to achieve scalable production of homogeneous organoids with high reproducibility. To validate the performance of the wp3D-OAHTS platform in handling ECM, we first compared the uniformity of cell-free ECM domes generated through manual seeding versus automated spotting. Manual dispensing of ECM exhibited significant inter-well variability in terms of dome position, volume, and morphology (Fig. 2A–C). In contrast, the automated spotter ensures precise central positioning within each well, and the size and shape of the ECM domes exhibited minimal inter-well variations (Fig. 2D–F), establishing a solid foundation for the generation of stable and reproducible 3D ECM-encapsulated organoids.

Next, we tested the organoid homogeneity cultured in our platform. Cervical cancer, gastric cancer, and colorectal cancer organoids were selected for validation due to their distinct morphological features, including solid, cyst-like, and budding structures. The organoids were dissociated into single cells, mixed with ECM, and processed by the platform for automated spotting and culture (with regular medium changes). Morphological changes and viability were monitored at 3-day intervals. The cell–ECM domes exhibited consistent size and shape uniformity. After 9 days of culture, all organoids displayed robust proliferation and maintained their characteristic structures (Fig. 2G–I). Viability measurements showed a narrow distribution, confirming the significantly reduced variability of organoids in our platform (Fig. 2J–L). These results demonstrate the capability of the platform in generating uniform, reproducible 3D ECM-encapsulated organoids with robust biological activity, qualifying it as a high-precision, reliable system for large-scale 3D organoid-based HTS.

### The wp3D-OAHTS platform enables 2802 drug screening on human NECC organoids

HTS based on human organoids is an effective strategy for drug discovery in rare diseases. Using the new platform and a drug library comprising 2802 small molecules, we analyzed the drug sensitivity of a patient-derived NECC organoid line we previously established. NECC is an aggressive histological subtype of cervical cancer accounting for about 1%–1.5% of all cervical cancers, with small cell neuroendocrine carcinomas being the most common form [25–30]. The prognosis of patients with NECC is poor, and there are few prospective studies or randomized trials specific to NECC to guide the standard of care management and therapy due to its rarity. Therefore, there is an urgent need to develop novel therapeutic agents and provide innovative treatment guidance for patients with NECC.

**Figure 3.**
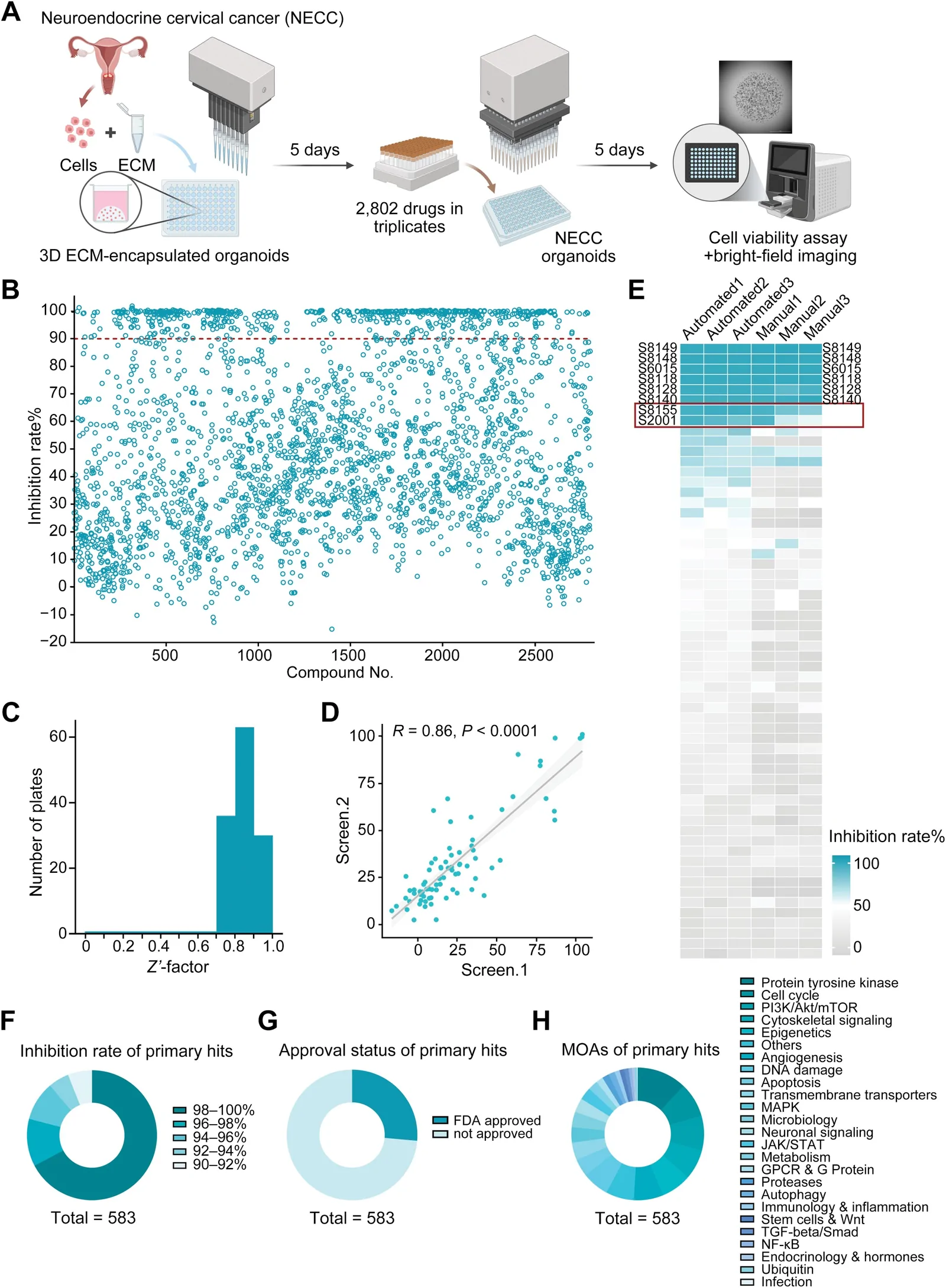
The wp3D-OAHTS platform enables 2802 drug screening on human NECC organoids. (A) Schematic workflow of conducting whole-process 3D NECC organoid drug screening using wp3D-OAHTS platform. (B) Scatter plot of the inhibition rates of 2802 compounds on NECC organoids, with 583 primary hits yielding over 90% inhibition rate tested at 10 μM. (C) Distribution of *Z′*-factors in 129 screening plates. (D) Correlation of the results between the different screen replicates using 77 randomly selected compounds. (E) Heatmap representation of inhibition rates for the compounds used to compare automated screening with manual screening. Positive results with inhibition rates greater than 90% are annotated on both sides. Two additional positive results identified by automated screening compared to manual screening, S8155 (RSL3) and S2001 (Elvitegravir), are highlighted in red boxes. (F–H) Pie plots displaying the distribution of inhibition rates (F), approval status (G), and mechanisms of action (H) among the 583 primary hits.

Using the wp3D-OAHTS platform, the dissociated single cells derived from NECC organoids were mixed with ECM and automatically spotted into 129 96-well plates in 3D ECM-encapsulated conditions (Fig. 3A). Consistent with other organoid models, the NECC organoids spotted and cultured by wp3D-OAHTS platform exhibited high homogeneity and robust proliferation (Fig. S2A and S2B). Five days later, 2802 compounds (10 μM) from libraries containing signaling pathway inhibitors, active pharmaceutical ingredients (APIs), natural products, and chemotherapeutic agents, were added to the plates. Cell viability was assessed after 5 days of treatment, and 583 compounds with inhibition rates of more than 90% were chosen as the primary hits (Fig. 3B). The *Z’*-factors were above 0.7 in all screening plates (Fig. 3C). The results from two independent assays of 77 randomly selected compounds revealed a high interexperiment correlation (Pearson’s coefficient of 0.86, *P* < 0.0001) (Fig. 3D). These results collectively illustrate the accuracy, robustness, and reproducibility of our drug screening platform.

In parallel, we performed a manual 3D organoid screening on a subset of the compounds. Interestingly, the automated screening platform identified eight hits, whereas the manual approach detected only six positive compounds (Fig. 3E). The manual screening failed to identify RSL3 (S8155) and Elvitegravir (S2001), primarily due to high variability among the three replicates treated with these two compounds, which led to unreliable average inhibition rates (Figs. 3E, S3A and S3B). In contrast, the automated screening platform achieved superior inter-replicate reproducibility, ensuring highly accurate and consistent results.

Among the 583 primary hits identified by the platform, more than half exerted lethal effects on NECC organoids (392 of 583), with inhibition rates ranging from 98% to 100% (Fig. 3F). The majority of the primary hits (429 of 583) were investigational compounds that have been evaluated in clinical trials or are in preclinical development, while only 154 compounds were approved by the Food and Drug Administration (FDA) (Fig. 3G). This fact suggests that there is a high probability that potential new NECC therapeutic candidates may emerge from compounds that have not yet received FDA approval. The 583 primary hits demonstrated a substantial degree of diversity in mechanisms of action (MOAs). Protein tyrosine kinase (PTK) inhibitors constituted the largest proportion at 12.18% (71 of 583), followed by cell cycle inhibitors and PI3K/Akt/mTOR inhibitors, each accounting for 8.40% (49 of 583) (Fig. 3H). These results indicate that high-throughput drug screening using 3D ECM-encapsulated organoids can reveal previously unidentified targets for rare diseases, providing a more diversified range of options for drug development.

### Reverse drug concentration escalation identifies seven top hits with potential for expanded indications in NECC

**Figure 4.**
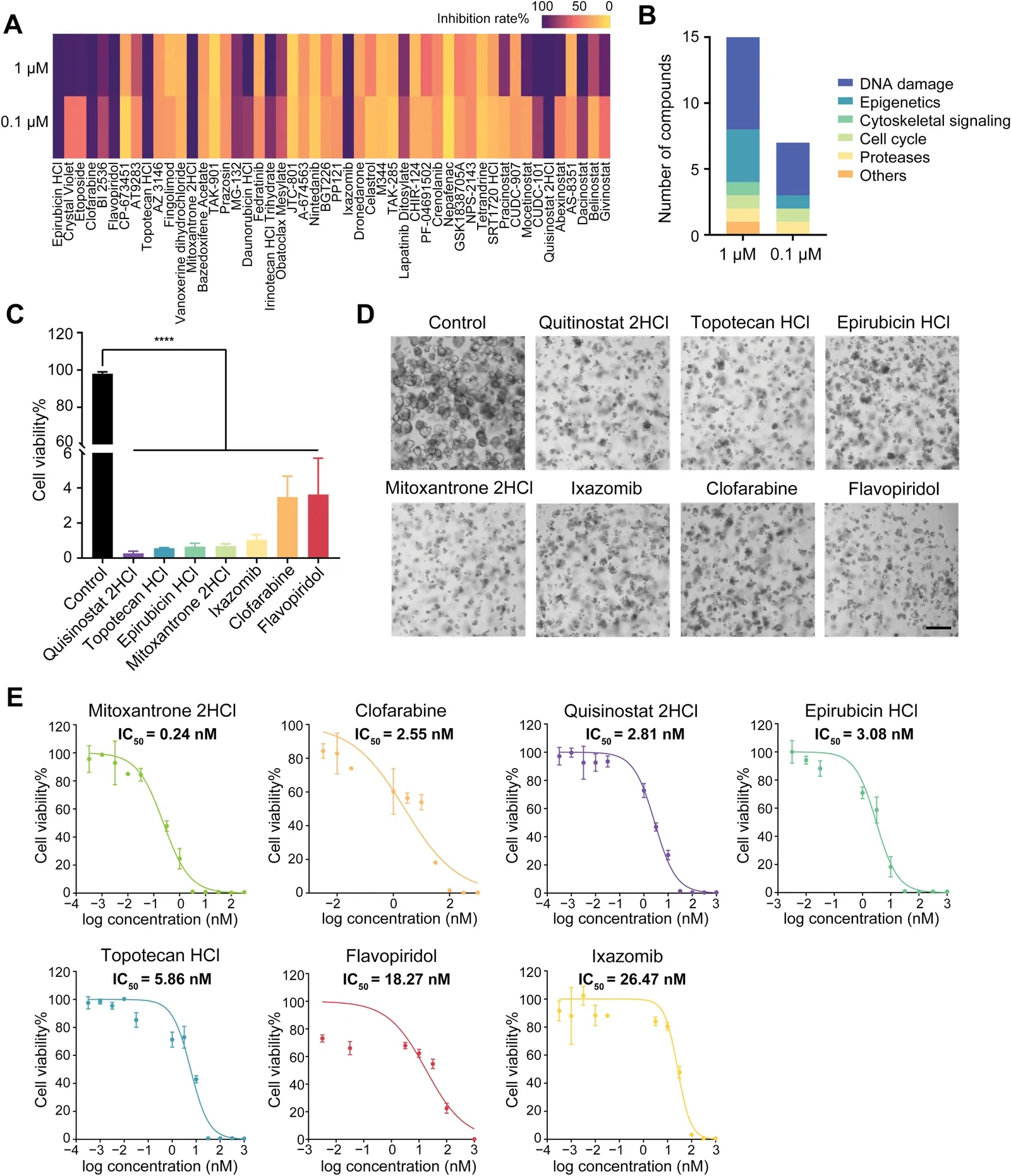
Reverse drug concentration escalation identifies 7 top hits with potential for expanded indications in NECC. (A) Heatmap representation of inhibition rates for NECC organoids in secondary screening versus the 50 compounds selected from primary screening using concentrations of 1 μM and 0.1 μM. 15 of them showed > 90% inhibition at 1 μM, and 7 of them were identified as top hits with > 90% inhibition at 0.1 μM. (B) Bar plots showing the distribution of mechanisms of action among the positive hits in secondary screening. (C) The normalized cell viability of NECC organoids after treatment with the 7 top hits at 0.1 μM. Data are presented as mean ± SD (*n* = 3). The *P* values were calculated by one-way ANOVA first and multiple comparisons test for further analysis. ****P < 0.0001. (D) Representative bright-field images of NECC organoids in the control group and after treatment with the 7 top hits at 0.1 μM. Scale bar, 200 μm. (E) Efficacy curves of the 7 top hits on NECC organoids. Data are presented as mean ± SD (*n* = 3).

To confirm and further narrow down the effective candidate drugs for NECC, we selected the top 50 compounds from our primary hits for a two-dose secondary screening (1 μM and 0.1 μM) against NECC organoids. Using the wp3D-OAHTS platform, we identified 15 and 7 candidates inducing growth inhibition > 90% at 1 μM and 0.1 μM, respectively (Fig. 4A). The 15 candidates identified at the 1 μM working concentration included inhibitors of DNA damage (7 of 15), epigenetics (4 of 15), cytoskeletal signaling (1 of 15), cell cycle (1 of 15), proteases (1 of 15), and others (1 of 15). Upon reducing the working concentration to 0.1 μM, 4 DNA damage inhibitors, 1 epigenetic inhibitor, 1 cell cycle inhibitor, and 1 protease inhibitor remained effective, emerging as the top hits (Fig. 4B). These 7 top hits, Quisinostat 2HCl, Topotecan HCl, Epirubicin HCl, Mitoxantrone 2HCl, Ixazomib, Clofarabine, and Flavopiridol, significantly reduced the viability of organoids and induced obvious cell death at 0.1 μM (Fig. 4C and 4D). To better understand their potency, we generated full effective 12-point dose curves for each compound (Fig. 4E). From this assay, all of the seven top hits were found to inhibit the growth of NECC organoids in a dose-dependent manner, with IC_50_ at the nanomolar level. Notably, five of these compounds (Mitoxantrone 2HCl, IC_50_ = 0.24 nM; Clofarabine, IC_50_ = 2.55 nM; Quisinostat 2HCl, IC_50_ = 2.81 nM; Epirubicin HCl, IC_50_ = 3.08 nM; and Topotecan HCl, IC_50_ = 5.86 nM) exhibited remarkably low IC_50_ below 10 nM, indicating their high potency and potential as promising therapeutic candidates for NECC.

Among the seven top hits, Mitoxantrone 2HCl, Clofarabine, Epirubicin HCl, Topotecan HCl, and Ixazomib are FDA-approved drugs, while Quisinostat 2HCl and Flavopiridol are compounds previously being investigated in clinical trials (Table S1). The current FDA-approved indications for these drugs do not include NECC, and the clinical trial data for Quisinostat 2HCl and Flavopiridol do not address NECC. These findings suggest their potential for expanding the therapeutic indications to include NECC.

### The representative drug Quisinostat 2HCl exhibits strong suppressive effects on NECC *in vivo*

To further validate the efficacy of the candidate drugs identified from our screening, we focused on the novel drug Quisinostat 2HCl, a non-FDA-approved compound with an IC_50_ of less than 10 nM. We examined the principal transcriptomic changes in NECC organoids treated with 0.1 μM Quisinostat 2HCl for 48 h and revealed a set of 3815 differentially expressed genes (DEGs; 1302 downregulated and 2513 upregulated, absolute foldchange ≥ 2 and *P*adj ≤ 0.05) (Fig. S4A and S4B). Gene set enrichment analysis (GSEA) identified significant downregulation in multiple metabolic pathways such as cholesterol metabolism, steroid synthesis, and glycolysis/gluconeogenesis in Quisinostat 2HCl-treated organoids (Fig. S4C–F). This finding suggests that Quisinostat 2HCl might inhibit the growth of NECC organoids by metabolism reprogramming.

**Figure 5.**
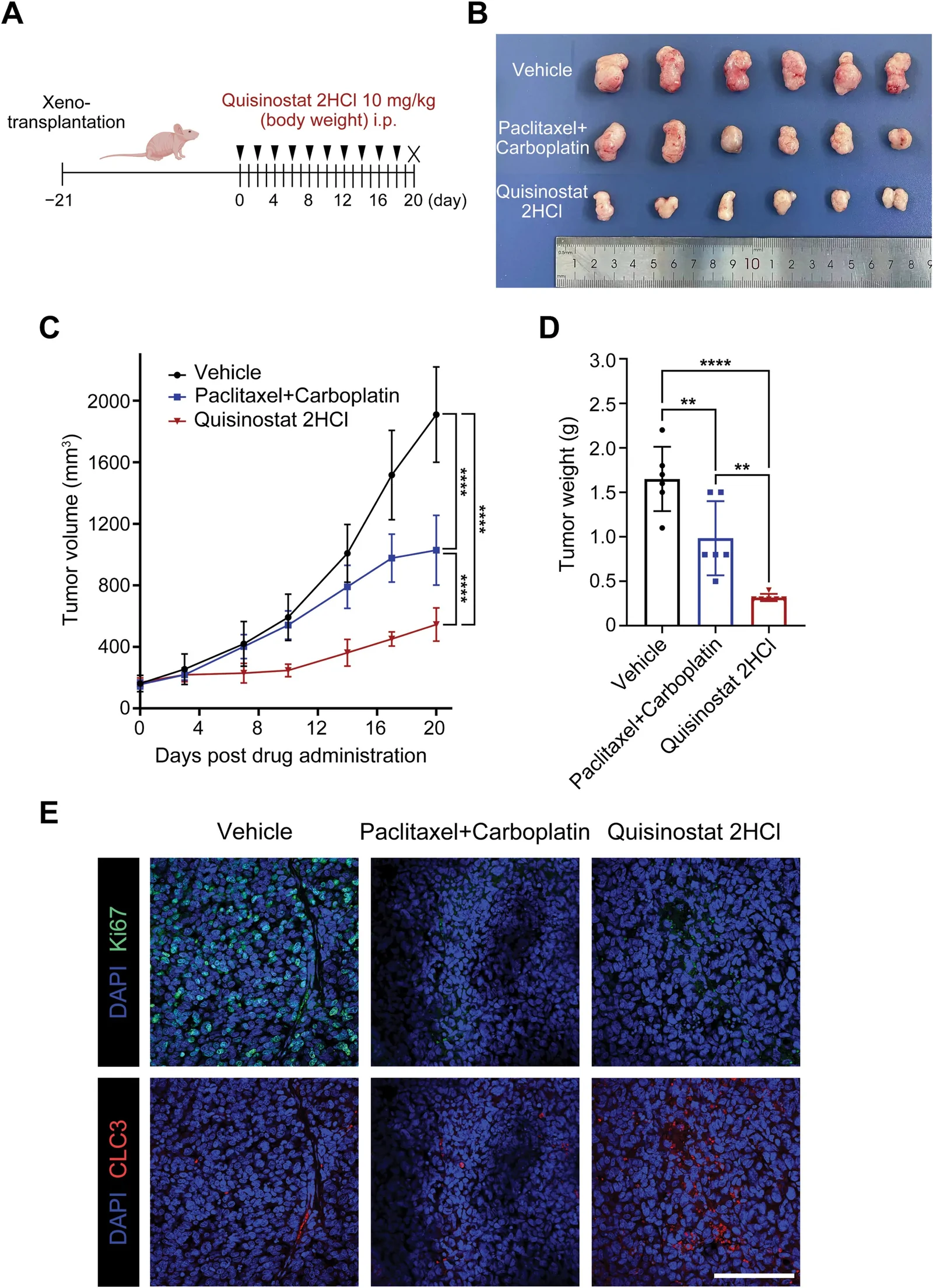
The representative drug Quisinostat 2HCl exhibits strong suppressive effects on NECC *in vivo*. (A) Schematic diagram of the NECC organoid xenograft model and *in vivo* drug treatment with Quisinostat 2HCl. Mice transplanted with NECC organoids received IP injections of 10 mg/kg Quisinostat 2HCl every other day for 20 days. (B) Image of mice harboring NECC organoid xenografts treated with Quisinostat 2HCl or Paclitaxel + Carboplatin or vehicle control. (C) Tumor growth curve of mice harboring NECC organoid xenografts treated with Quisinostat 2HCl or Paclitaxel + Carboplatin or vehicle control. Data are presented as mean ± SD. *n* = 6 mice. (D) Tumor weight of mice harboring NECC organoid xenografts treated with Quisinostat 2HCl or Paclitaxel + Carboplatin or vehicle control. Data are presented as mean ± SD. *n* = 6 mice. (E) Representative Ki67 and cleaved caspase 3 (CLC3) staining of NECC organoid xenografts treated with Quisinostat 2HCl or Paclitaxel + Carboplatin or vehicle control. Scale bar, 100 μm. The p values were calculated by one-way ANOVA first and multiple comparisons test for further analysis. For tumor growth curve, the *P* values were calculated by two-way ANOVA. **P < 0.01, ***P < 0.001, and ****P < 0.0001.

To evaluate the anti-tumor effects of Quisinostat 2HCl *in vivo*, NECC organoids were transplanted subcutaneously in nude mice. When the transplanted tumors reached the designated volume, the mice were treated with 10 mg/kg Quisinostat 2HCl via intraperitoneal (IP) injection every other day for 20 days (Fig. 5A) [31]. The combination treatment of paclitaxel and carboplatin was included due to its established clinical application in the treatment of NECC and the favorable therapeutic effect observed in our NECC patient [32]. The growth of NECC xenografts in Quisinostat 2HCl-treated mice was significantly impaired compared with vehicle-treated mice, as evidenced by a marked decrease in both tumor volume and weight (Fig. 5B–D). Quisinostat 2HCl significantly decreased the percentage of Ki67^+^ cells and increased the percentage of cleaved caspase3^+^ (CLC3^+^) cells in xenografts (Fig. 5E). It is notable that Quisinostat 2HCl treatment exhibited a higher inhibitory efficiency than the combination treatment of paclitaxel and carboplatin, which indicates its potential superiority as a monotherapy for NECC. These results collectively demonstrate that Quisinostat 2HCl possesses a potent capacity to inhibit the growth of NECC *in vivo*, highlighting its potential as a therapeutic agent for the treatment of NECC.

### The wp3D-OAHTS platform permits precise drug responses by restricting false positives caused by organoid suspension

We have illustrated that cultivation of organoids in a matrix-low suspension condition can induce extensive transcriptomic alterations (Fig. S1). To further investigate whether these changes in culture conditions influence the outcomes of organoid drug screening, we selected 6–7 compounds from each of the following inhibition rate ranges between 90%–100%, 70%–80%, 50%–60%, and 30%–40%, based on the initial 3D ECM-encapsulated organoid drug screening of 2802 small molecules. These compounds were then tested against NECC organoids suspended in matrix-low conditions.

**Figure 6.**
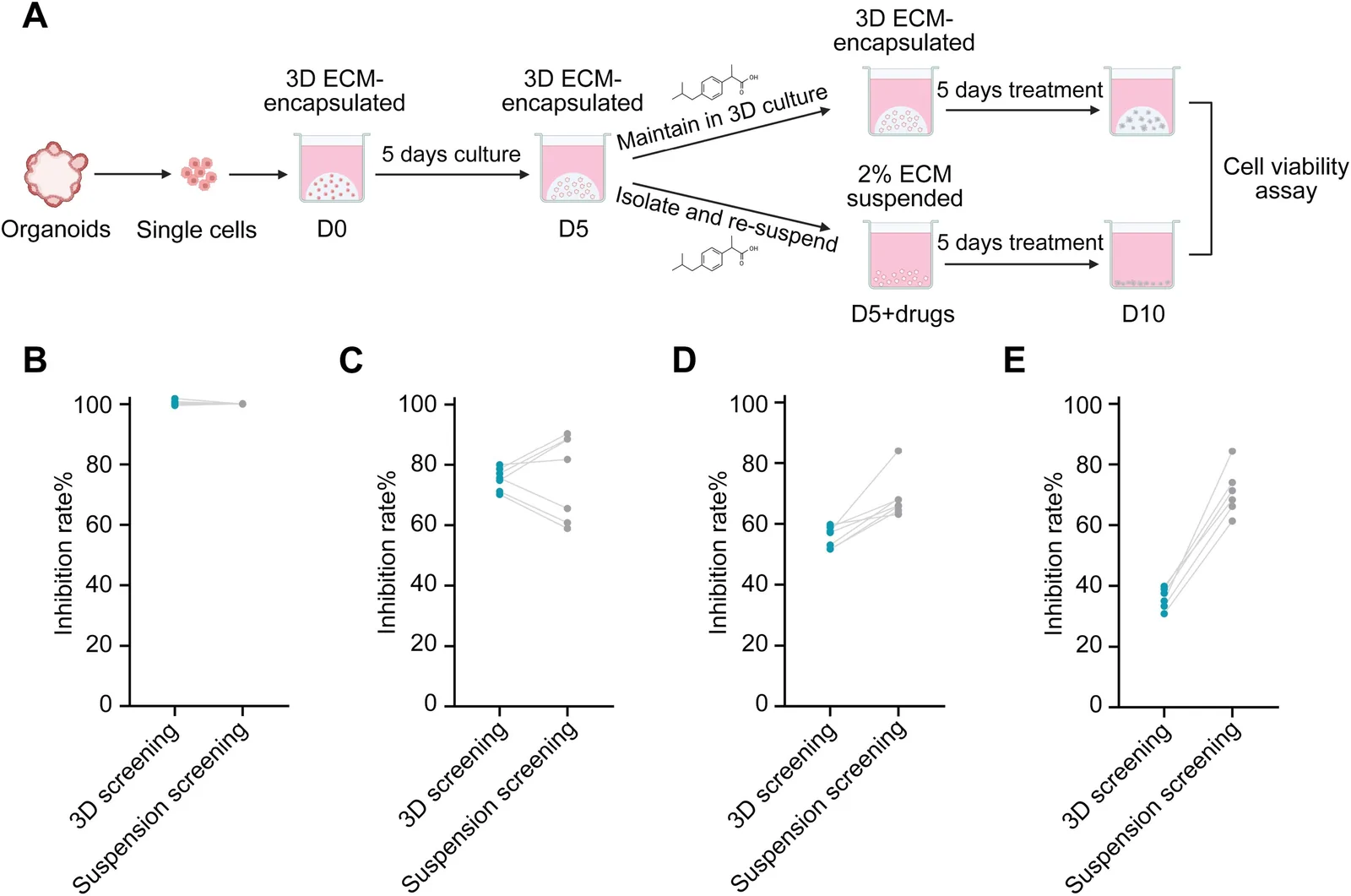
The wp3D-OAHTS platform permits precise drug responses by restricting false positives caused by organoid suspension. (A) Schematic workflow of whole-process 3D ECM-encapsulated organoid-based screening and organoids screening suspended in matrix-low conditions. (B–E) Changes in inhibition rate of NECC organoids in 3D screening (left) and suspension screening (right). Each dot represents one compound.

The conventional suspension-based organoid screening is similar to the whole-process 3D ECM-encapsulated organoid screening described here in that its initial phase involves encapsulating dissociated single cells in ECM, seeding them into wells of culture plates, and adding culture medium after gelation [12]. However, on the day of drug application, organoids in the suspension screening are detached from the ECM and resuspended in a culture medium containing 2% (*v*/*v*) ECM. After that, organoids are deprived of the supportive 3D environment and instead reside within a non-adherent suspension condition. Following 5 days of treatment with specific compounds, cell viability is assessed (Fig. 6A).

For small molecules exhibiting high sensitivity in NECC organoids, the outcomes between the whole-process 3D screening and suspension screening showed little discrepancy (Fig. 6B). Nevertheless, compounds that were previously shown to be non-responsive in the whole-process 3D screening exhibited enhanced sensitivity under suspension conditions. We found that over half of the small molecules with inhibition rates between 70% and 80% in 3D screening exhibited increased inhibition rates in suspension screening (Fig. 6C). Furthermore, all small molecules with inhibition rates between 50% and 60% in 3D screening showed increased inhibition rates in suspension screening (Fig. 6D). Meanwhile, those with inhibition rates between 30% and 40% in 3D screening also displayed inhibition rates with a more remarkable increase (Fig. 6E). This manifests that downgrading the 3D-cultured organoids to adapt to a 2D suspension system significantly increases the hit rate in organoid-based drug screening. However, these positive results may not accurately reflect the drug responses and resistance patterns of *in vivo* tissues, possibly attributed to the alterations in the 3D culture environment and transcriptomic landscape [33]. Applying 3D ECM-encapsulated organoids for drug screening enables the preservation of *in vivo*-like 3D structure of organoids, which is fundamental for the organoids to recapitulate drug responses of *in vivo* tissues, thereby fully leveraging the value of organoids in high-throughput drug screening.

## Discussion

Here, we present a robust wp3D-OAHTS platform capable of 3D ECM-encapsulated organoids automated spotting, culturing, monitoring, and HTS in 96-well plates. Organoids are maintained under 3D culture conditions encapsulated in an ECM component, which allows drug screening to be carried out in a state that most closely mimics the real tissues *in vivo*. Utilizing patient-derived 3D organoids for *ex vivo* drug screening has become a widely accepted practice. However, most HTS studies involving organoids were conducted in matrix-free or matrix-low culture medium due to challenges associated with automated HTS in ECM-encapsulated formats [1, 3, 12, 34, 35]. To circumvent these problems, researchers often choose to downgrade 3D organoid cultures to adapt to conventional, simple HTS devices. In this study, we have shown that even shorter durations of culture in matrix-low conditions can lead to widespread alterations in the organoid transcriptome, leading to modifications in their drug responses (Figs. 6 and S1). Such strategies diminish the inherent advantages of organoids and make it difficult to reflect the true drug responses.

Previous studies have reported several methods for generating 3D ECM-encapsulated organoids for HTS. (1) Printing cell–matrix mixtures in mini-rings or mini-squares with empty centers in microwell plates [15, 16]. The empty center architecture facilitates medium exchanges and drug addition and can be adapted to traditional automation devices. However, this method requires more dispensing time compared to generating domes in wells due to the need for specific geometries. (2) Utilizing microfluidic systems to create uniform cell–matrix droplets or micro-organospheres [17, 18]. These platforms generate organospheres using various separation oils, which introduce additional steps for droplet washing and oil removal before dispensing. This method ensures uniformity but increases the complexity of the process. (3) Spotting cell–matrix mixtures onto the micropillar surface [19–22], and inverting the micropillars onto microwell plates containing medium or drugs. This method requires specialized micropillar plates and additional coating with poly-l-lysine to enhance adhesion between droplets and the micropillar surface. To overcome the limitations described above, our wp3D-OAHTS platform is designed to automatically spot cell–matrix mixtures into the center of wells in 96-well plates, simulating the traditional manual organoid culturing process but with superior stability and reproducibility. This approach does not require the introduction of additional consumables or steps, thus significantly simplifying the workflow, reducing processing time, and improving screening throughput.

We utilized a patient-derived organoid line from NECC, a rare malignancy with significant unmet clinical needs for novel drug development [25–27], to perform HTS of 2802 small molecules on our platform, to demonstrate the effectiveness, robustness, reproducibility, and stability of our screening system. We developed a two-step tiered HTS approach composed of primary screening and secondary screening, identifying 7 top hits from 2802 drugs that achieved over 90% inhibition rate of organoids at a working concentration of 0.1 μM. Five of the seven top hits have an IC_50_ of less than 10 nM, suggesting a potential clinical application in NECC treatment. Furthermore, we selected Quisinostat 2HCl, the only preclinical drug among these five candidates that has not yet received FDA approval, for *in vivo* efficacy validation. We found that Quisinostat 2HCl effectively inhibited the growth of NECC xenografts in mice. Quisinostat is an orally bioavailable potent pan-histone deacetylase inhibitor that has been tested in phase II clinical trials for cutaneous T-cell lymphoma and ovarian cancer but has not previously been reported to have any effect on neuroendocrine neoplasms [36, 37]. Our *in vitro* and *in vivo* results suggest that Quisinostat 2HCl has great potential to be a therapeutic agent for neuroendocrine neoplasms treatment.

Compared to manual seeding of organoids in 96-well plates for drug screening, our automated platform produces more consistent ECM domes across wells, contributing to a higher stability between replicate wells during drug screening. For example, in comparative testing of the manual and the automated systems, RSL3 and Elvitegravir were classified as positive hits by the automated screening but classified as negative hits by the manual screening. They accounted for 25% of the positive hits identified by the automated system. Based on bright-field imaging records, we observed significant variability between the replicate wells in the manual screening with inconsistent responses to drug treatment (Fig. S2). In contrast, the automated screening demonstrated higher reproducibility across the three replicate wells. Adopting automated screening instead of manual screening can avoid the loss of 25% of positive results, which emphasizes the automation of HTS.

Additionally, we compared the drug screening results of organoids cultured in suspension to those cultured in 3D conditions. We found that suspension cultures could increase organoid sensitivity to drugs, particularly for those that were insensitive under 3D culture conditions (Fig. 6). The finding may be attributed to the loss of 3D support in suspension culture, leading to a deviation from the *in vivo* tissue state and a shift towards a 2D-like condition. This possibly results in false positive outcomes similar to those obtained with 2D cell line screens and a high failure rate during clinical trials. Thus, compared to manual and suspension screening, our 3D ECM-encapsulated organoid-based automated HTS demonstrates superior stability and efficacy, making it a definitive choice for organoid drug screening.

**Figure 7.**
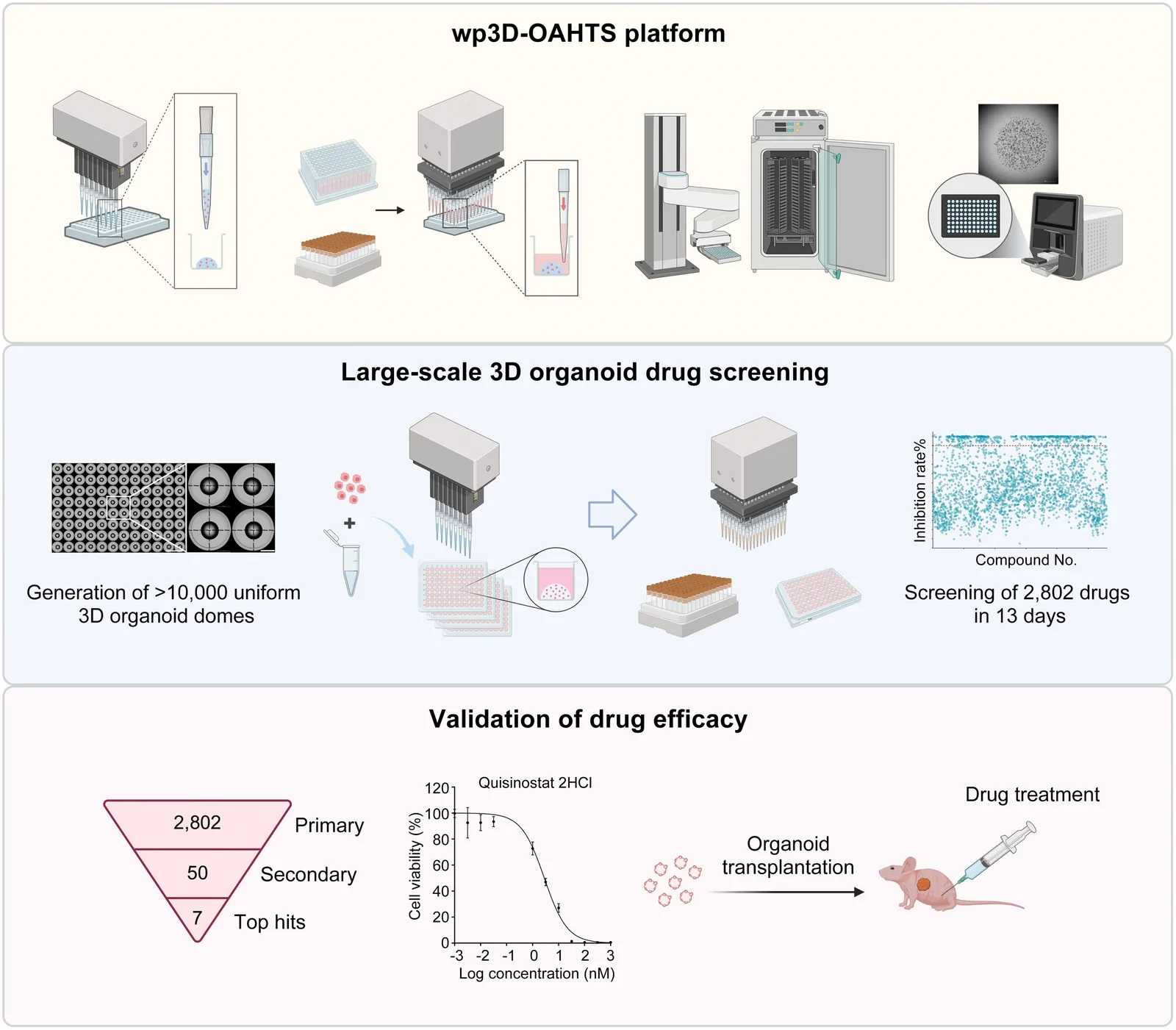
Graphical summary.

In summary, to maximize the advantages of 3D organoids in accurately reflecting *in vivo* tissue drug responses, we have developed an automated organoid HTS platform that allows rapid generation of large-scale 3D organoids and automated evaluation of their drug responses. This platform achieves a more homogeneous, precise, and high-throughput seeding of 3D organoids compared to manual operation. Based on this, we have conducted a two-step tiered high-throughput drug screening of 2802 small molecules using 3D organoids for NECC, a rare malignancy with an urgent clinical need for new therapeutic options. We have identified seven candidate drugs with extremely low IC_50_, and we have validated the *in vivo* anti-tumor effect of one of these drugs, Quisinostat 2HCl. Furthermore, the wp3D-OAHTS platform allows for precise drug responses by restricting false positives caused by suspension cultures of organoids. These findings collectively demonstrate the superiority of using 3D ECM-encapsulated organoids for high-throughput drug screening over the conventional manual system or the suspension system. The application of the wp3D-OAHTS platform would significantly improve the rapidity and efficiency of new drug discovery for rare diseases (Fig. 7).

## Research limitations

This study also has several limitations. Although, this platform permitted a proof-of-concept screening of 2802 drugs, future advancements in instrumentation to enable high-precision spotting in 384-well plates would significantly enhance the throughput of 3D ECM-encapsulated organoid-based screening. Besides, more experiments are needed to elucidate the mechanism by which Quisinostat 2HCl regulates NECC, which could deepen our understanding of its therapeutic potential.

## Methods

### wp3D-OAHTS platform design and assembly

The wp3D-OAHTS platform used for 3D ECM-encapsulated organoid-based HTS was designed and specified by our team at Fudan University, and subsequently customized and supplied by Novogene. This platform comprises four main modules: a 3D ECM-encapsulated organoids spotter, a liquid dispensing module, an automated incubating module, and a multimode-detecting module. The detailed sub-modules include a cooling unit, an 8-channel ECM spotting head, a heating-shaking unit, a 96-channel liquid dispensing head, a plate transfer unit, incubators, a robotic arm, a multimode reading unit, an imaging unit, a customized gripper unit, an automated plate storage unit, a workstation platform, and a laminar flow hood.

### Research ethics

In this study, the NECC patient provided written informed consent to participate in the clinical trial, in accordance with the principles of the Helsinki Declaration. The NECC biopsies and organoid establishment were conducted with the approval of the Medical Ethics Committee of Shanghai Chest Hospital (approval number: KS(Y)2078).

All animal experiments were approved by the Institutional Animal Care and were compliant with all relevant ethical regulations regarding animal research. The animal ethics (2020JS038) was approved by the Laboratory Animal Center, School of Life Sciences, Fudan University.

### Organoid culture

The NECC, CC, GC, and CRC organoids were grown in drops of Organoid Culture ECM (bioGenous, M315066) in 24-well plates, overlaid with the corresponding medium. NECC organoids were maintained in Advanced DMEM/F-12 supplemented with penicillin–streptomycin (Invitrogen), HEPES buffer (10 mM, Gibco), GlutaMax (1×, Invitrogen), B27 supplement (1×, Invitrogen), N-acetyl-L-cysteine (1.25 mM, Sigma-Aldrich), Nicotinamide (5 mM, Sigma-Aldrich), A-83-01 (500 nM, bioGenous), SB202190 (500 nM, bioGenous), Y-27632 (5 µM, bioGenous), R-spondin1 (500 ng/mL, bioGenous), Noggin (100 ng/mL, bioGenous), EGF (50 ng/mL, bioGenous), FGF10 (100 ng/mL, bioGenous), and FGF7 (25 ng/mL, bioGenous). Cervical Cancer Organoid Kit (bioGenous, K2169-CC) and Gastric Cancer Organoid Kit (bioGenous, K2179-GC) were used for the expansion of CC organoids and GC organoids, respectively. CRC organoids were maintained in Advanced DMEM/F-12 supplemented with penicillin–streptomycin (Invitrogen), GlutaMax (1×, Invitrogen), B27 supplement (1×, Invitrogen), N-acetyl-L-cysteine (1 mM, Sigma-Aldrich), Nicotinamide (10 mM, Sigma-Aldrich), A-83-01 (500 nM, bioGenous), SB202190 (3 µM, bioGenous), R-spondin1 (500 ng/mL, bioGenous), Noggin (100 ng/mL, bioGenous), EGF (50 ng/mL, bioGenous), Gastrin-1 (100 µM, bioGenous), and PGE2 (1 nM, bioGenous). The medium was changed every 3–4 days.

### Organoid growth assessment

The growth of NECC, CC, GC, and CRC organoids after being automatically seeded by the wp3D-OAHTS platform was quantified by an increase in cell viability. Organoids grown in 20 μL ECM domes in 24-well plates were released from ECM in cold PBS, followed by centrifugation at 300 *g* for 3 min and resuspension in 1 mL of organoid dissociation solution (bioGenous, E238001). The organoids were digested at 37°C until they became single cells and were filtered using a 40-μm pore cell strainer. The filtrate was centrifuged at 300 *g* for 3 min. The cells were then mixed with culture medium and ECM (ratio = 1:4) at the appropriate density and added to the wells of the sample plate, which was kept at 0°C by the cooling unit. The cell–ECM mixtures (3 μL per well) were spotted into the centers of each well in a 96-well plate (Corning 3603 clear black flat-bottom microplates) using the 3D ECM-encapsulated organoids spotter. The plates were heated at 37°C on the heating unit for 5 min for gelation. 100 μL of 1 × LivingCell-Fluo™ Organoid Vitality Assay (bioGenous, E238004) reagent was added into each well and incubated in a 37°C incubator for another 30 min. Fluorescence was measured at excitation 560 nm and emission 590 nm. Cell viability was detected and imaged every 3 days.

### Organoid drug screening on the wp3D-OAHTS platform

A compound library containing small molecular inhibitors and clinically used drugs was obtained from Selleck. NECC organoids grown in 20 μL ECM domes in 24-well plates were harvested, digested, and spotted into the 96-well plates as described above. Following gelation at 37°C on the heating unit for 5 min, 100 μL of culture medium was dispensed into each well using a liquid dispensing head. The plates were then transferred to the detector for organoid imaging via a robotic arm before being returned to the incubators for further culture. Throughout the drug screening phase, each organoid plate was retrieved daily by the robotic arm from the incubators and transferred to the detector for imaging and monitoring, providing daily updates on the organoid growth status. After 5-day post-seeding of organoids, the automated liquid dispensing head performed the dosing procedure. Inhibitors were introduced into the culture medium at the final concentration of 10 μM during primary screening, while the 50 selected hits underwent secondary screening at final concentrations of 1 μM and 0.1 μM. For dose curve confirmation, the seven top hits were added to the culture medium at different final concentrations. Positive and negative controls were also included. After 5 days of treatment, the plates were retrieved by the robotic arm and imaged using the detector, followed by placement in the liquid dispensing module for medium removal. 100 μL of 1 × LivingCell-Fluo™ Organoid Vitality Assay (bioGenous, E238004) reagent was added into each well and incubated in a 37°C incubator for another 30 min. Fluorescence was measured at excitation 560 nm and emission 590 nm. To determine the percentage reduction in treated organoids versus control organoids in cytotoxicity assays, the following formula was used:

Inhibition rate (%) = 100 − (FI_compound_ − FI_positive_)/(FI_negative_ − FI_positive_) × 100

Where FI = Fluorescence intensity.

To calculate IC_50_, viability was normalized relative to the average of positive controls and the highest concentration of drug-treated conditions. Efficacy curves were generated using Prism GraphPad.

### Drug validation in xenograft model

Six-week BALB/c-Nude mice (Strain NO. D000521) were purchased from GemPharmatech (Nanjing, China). Organoids were transplanted subcutaneously with 100,000 cells in 100 μL 50% ECM per injection in PBS. Tumor volume was measured with calipers. After the tumor volume of each mouse reached 150–200 mm^3^, the mice were randomly divided into three groups for treatment with drugs or vehicles. Quisinostat 2HCl was administered at 10 mg/kg via intraperitoneal injection every other day. Combination therapy of paclitaxel (10 mg/kg) with carboplatin (50 mg/kg) was administered via intraperitoneal injection once a week. Mice were sacrificed 20 days after drug administration.

### Immunofluorescence staining

Tissues were fixed with 4% paraformaldehyde and embedded in paraffin. Sections were deparaffinized in xylene and graded alcohols, followed by antigen retrieval. Sections were then blocked with 5% BSA in PBS for 1 h at room temperature, and incubated overnight at 4°C with primary antibodies. The following antibodies were used: anti-Ki67 (BD Biosciences,1:400) and anti-cleaved-caspase3 (CST, 1:400). The respective secondary antibodies (Abbkine, 1:400) were incubated at room temperature for 1 h. Images were taken by confocal microscopy (Leica DMI8).

### RNA-seq

Total RNA of NECC organoids suspended in culture medium containing 2% (*v*/*v*) ECM, NECC organoids encapsulated in 3D ECM, and NECC organoids treated with 0.1 μM Quisinostat 2HCl or PBS for 48 h were isolated using the RNAprep Pure Micro Kit (Tiangen Biotech) and then sent for sequencing (Novogene). High-throughput sequencing was performed using Illumina NovaSeq X Plus for three biological replicates, respectively. The raw sequencing data quality was checked by FastQC. HISAT2 was then used to align clean reads to the human reference genome (GRCh38) with default parameters. Bam files were sorted by Samtools and count matrices were generated by StringTie.

Downstream analysis was processed in R v4.2.1. Briefly, DESeq2 v1.38.3 was used to identify DEGs. For comparison between NECC organoids suspended in culture medium containing 2% ECM and encapsulated in 3D ECM, genes with padj ≤ 0.05 and absolute foldchange ≥ 1.5 were regarded as DEGs. For comparison between NECC organoids treated with 0.1 μM Quisinostat 2HCl or PBS, genes with padj ≤ 0.05 and absolute foldchange ≥ 2 were regarded as DEGs. ClusterProfiler v4.6.2 was used to do KEGG enrichment and GSEA, and gene expression heatmaps were generated by pheatmap v 1.0.12.

### Statistical analysis

We employed one-way ANOVA test and two-way ANOVA test to analyze the experimental results. Analyses were conducted on GraphPad Prism 8 statistical software. All values are represented as means ± SD. The value of *P < 0.05, **P < 0.01, ***P < 0.001, and ****P < 0.0001 was considered significant.

## Supplementary Material

lnaf021_suppl_Supplementary_Materials

lnaf021_suppl_Supplementary_Table

## Data Availability

RNA-seq data have been deposited at NCBl SRA database and are publicly available with the accession number: PRJNA1261448 and PRJNA1261613.
