# Supplementary material for: Whole-process 3D ECM-encapsulated organoid-based automated high-throughput screening platform accelerates drug discovery for rare diseases
Source: Life Med. 2025 Jun 14;4(5):lnaf021. doi: 10.1093/lifemedi/lnaf021 (PMC12517169; doi:10.1093/lifemedi/lnaf021)
Supplement: lnaf021_suppl_Supplementary_Materials [file lnaf021_suppl_supplementary_materials.docx]

**Whole-Process 3D ECM-Encapsulated Organoid-Based Automated High-Throughput Screening Platform Accelerates Drug Discovery for Rare Diseases**

**Zhaoting Xu^1^, Hui Yang^2^, Yuru Zhou^3^, Emmanuel Enoch Dzakah^4^, Bing Zhao^2,3,4,^***

^1^State Key Laboratory of Genetic Engineering, School of Life Sciences, Fudan University, Shanghai 200438, China

^2^Institute of Organoid Technology, Kunming Medical University, Kunming 650500, China

^3^School of Basic Medical Sciences, Institute of Biomedical Innovation, The First Affiliated Hospital, Jiangxi Medical College, Nanchang University, Nanchang 330031, China

^4^Z Lab, bioGenous BIOTECH, Shanghai 200438, China

*Correspondence: bingzhao@fudan.edu.cn (B.Z.)

**Supplementary Figure S1**


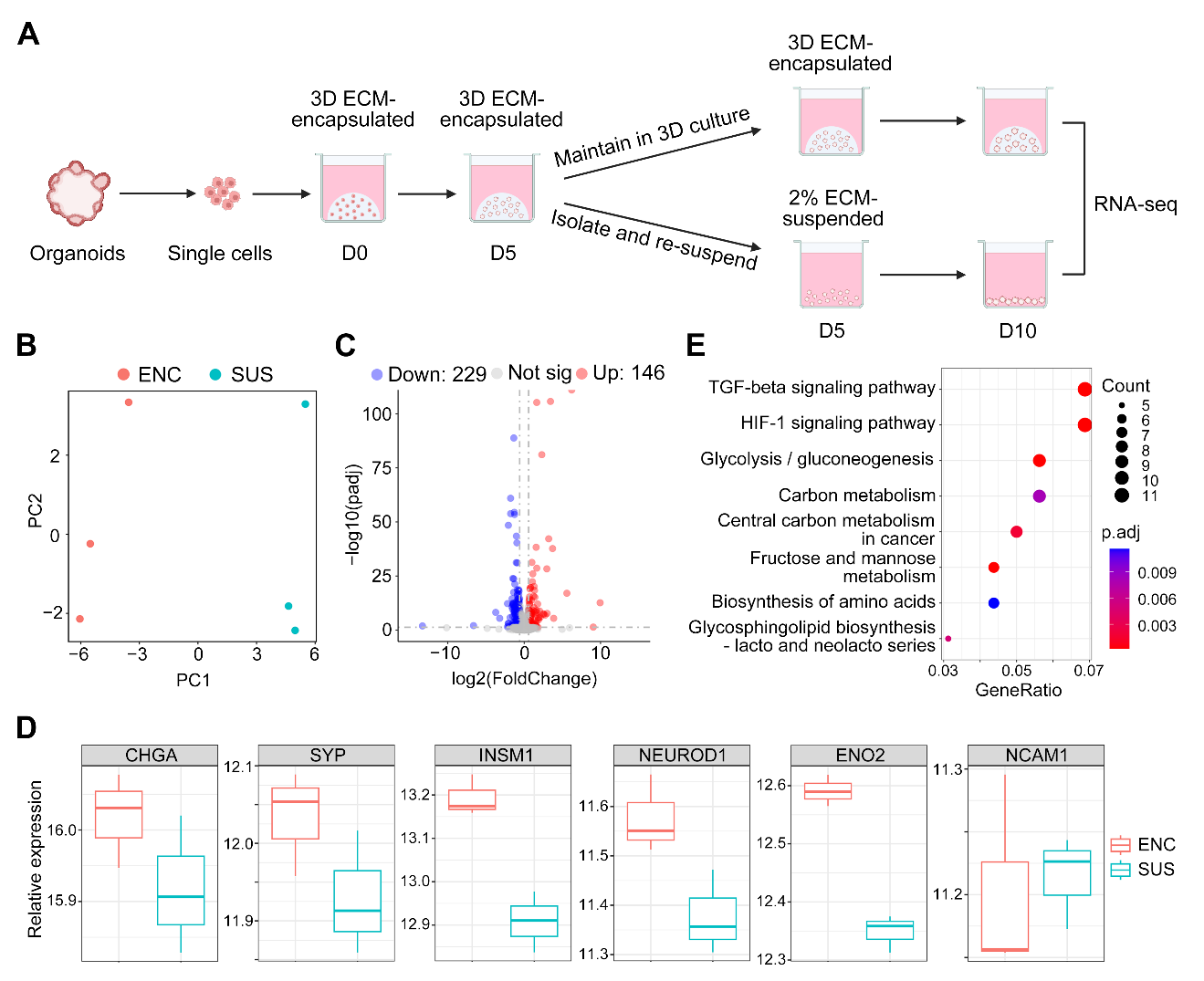


**Supplementary Figure S1. Traditional organoid drug screening suspended in matrix-low conditions induces transcriptomic modifications.**

(A) Schematic workflow of RNA-seq for whole-process 3D ECM-encapsulated organoid cultures and traditional suspended organoid cultures. (B) Principal component analysis of encapsulated (*n* = 3) and suspended (*n* = 3) organoids. ENC, encapsulated organoids. SUS, suspended organoids. PC, principal component. (C) Volcano plot of differentially expressed genes (DEGs) in suspended versus encapsulated organoids. Genes with padj ≤ 0.05 and absolute foldchange ≥ 1.5 were regarded as DEGs. (D) Boxplot of the relative expression of tumor markers in encapsulated and suspended organoids. (E) KEGG analysis of DEGs in suspended organoids, compared to encapsulated organoids.

**Supplementary Figure S2**


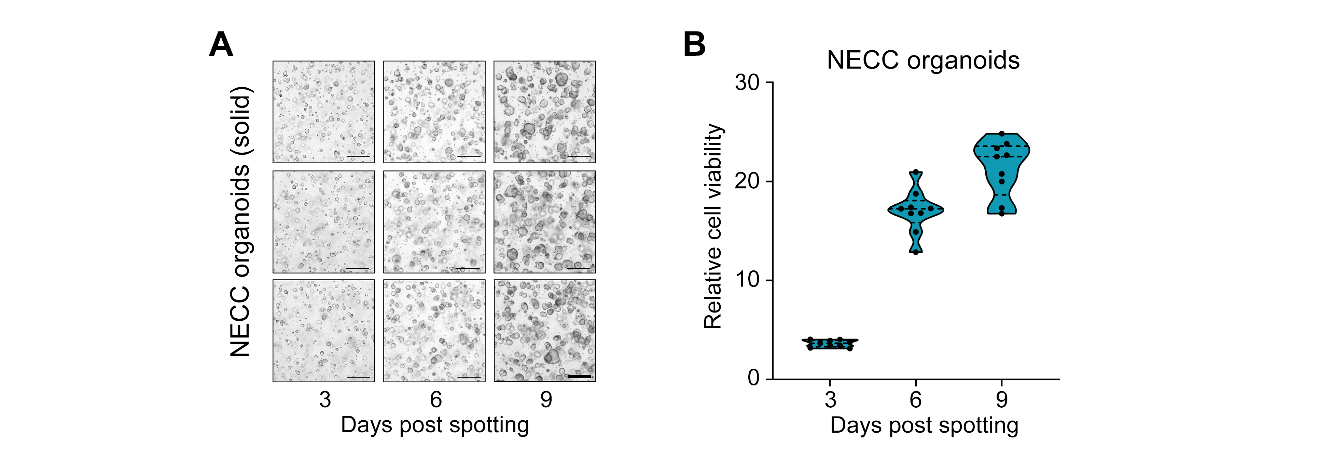


**Supplementary Figure S2. NECC organoids spotted and cultured by wp3D-OAHTS platform exhibits high homogeneity and robust proliferation.**

(A) Representative bright-field images showing NECC organoids growth after being automatically spotted and cultured by wp3D-OAHTS platform. Scale bar, 200 μm. (B) Violin plots of cell viability change ratio of NECC organoids after being automatically spotted and cultured by wp3D-OAHTS platform.

**Supplementary Figure S3**


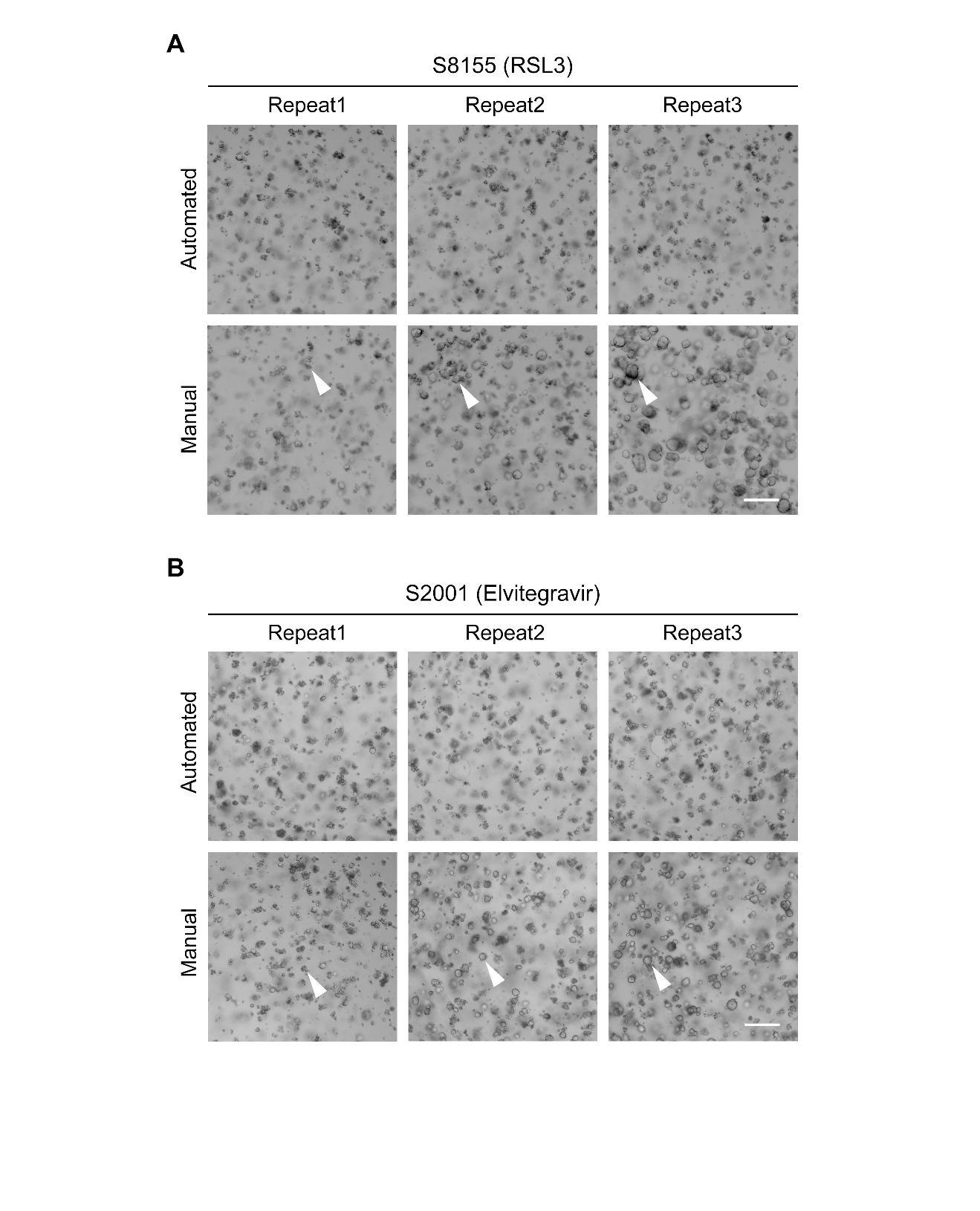


**Supplementary Figure S3. Automated 3D screening exhibits higher stability compared to manual 3D screening and recovers positive hits missed by manual operation.**

(A) Bright-field images of organoids in three replicate wells for automated (top) and manual (bottom) screening after treatment with S8115 (RSL3) at 10 μM. Scale bar, 200 μm. White arrows indicate the differences in organoid growth in the three replicate wells of the manual screening. (B) Bright-field images of organoids in three replicate wells for automated (top) and manual (bottom) screening after treatment with S2001 (Elvitegravir) at 10 μM. Scale bar, 200 μm. White arrows indicate the differences in organoid growth in the three replicate wells of the manual screening.

**Supplementary Figure S4**


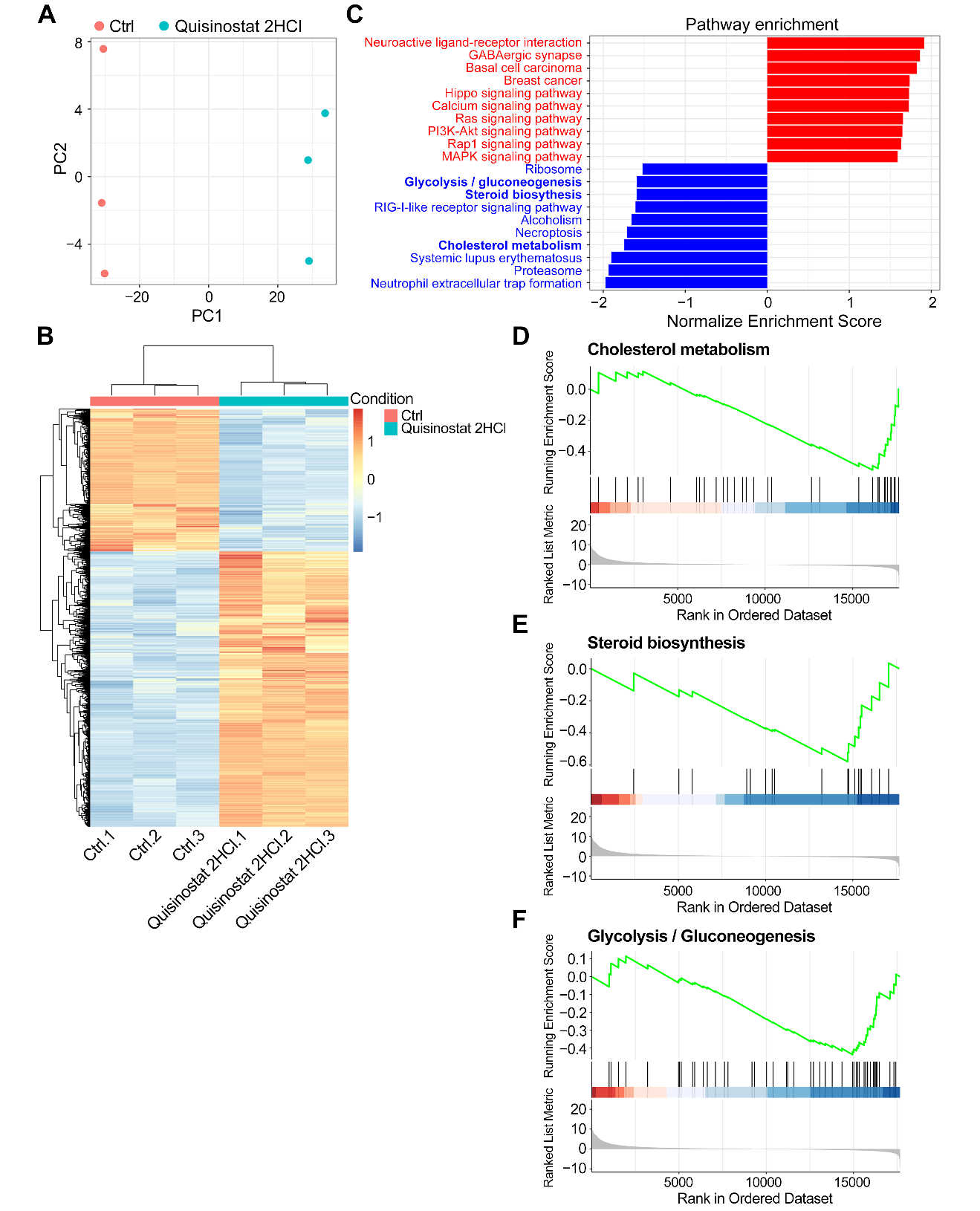


**Supplementary Figure S4. Quisinostat 2HCl might inhibit the growth of NECC organoids by metabolism reprogramming.**

(A) Principal component analysis of control (*n* = 3) and Quisinostat 2HCl-treated (*n* = 3) organoids. PC, principal component. (B) mRNA expression heatmap of differentially expressed genes for control (*n* = 3) and Quisinostat 2HCl-treated (*n* = 3) organoids. (C) Gene set enrichment analysis (GSEA) of Quisinostat 2HCl-treated versus control organoids. Upregulated pathways are shown in red text, and downregulated pathways are shown in blue text. (D–F) GSEA analysis showing enrichment of cholesterol metabolism, steroid biosynthesis, and glycolysis/gluconeogenesis pathway in Quisinostat 2HCl-treated versus control organoids.
